# A critical evaluation of protein kinase regulation by activation loop autophosphorylation

**DOI:** 10.7554/eLife.88210

**Published:** 2023-07-20

**Authors:** Ronja Reinhardt, Thomas A Leonard

**Affiliations:** 1 Max Perutz Labs, Vienna Biocenter Campus (VBC) Vienna Austria; 2 Medical University of Vienna, Center for Medical Biochemistry Vienna Austria; Walter and Eliza Hall Institute of Medical Research Australia; Goethe University Germany

**Keywords:** kinase, allostery, inhibition, activation, phosphorylation, dimerization

## Abstract

Phosphorylation of proteins is a ubiquitous mechanism of regulating their function, localization, or activity. Protein kinases, enzymes that use ATP to phosphorylate protein substrates are, therefore, powerful signal transducers in eukaryotic cells. The mechanism of phosphoryl-transfer is universally conserved among protein kinases, which necessitates the tight regulation of kinase activity for the orchestration of cellular processes with high spatial and temporal fidelity. In response to a stimulus, many kinases enhance their own activity by autophosphorylating a conserved amino acid in their activation loop, but precisely how this reaction is performed is controversial. Classically, kinases that autophosphorylate their activation loop are thought to perform the reaction in *trans*, mediated by transient dimerization of their kinase domains. However, motivated by the recently discovered regulation mechanism of activation loop *cis*-autophosphorylation by a kinase that is autoinhibited in *trans*, we here review the various mechanisms of autoregulation that have been proposed. We provide a framework for critically evaluating biochemical, kinetic, and structural evidence for protein kinase dimerization and autophosphorylation, and share some thoughts on the implications of these mechanisms within physiological signaling networks.

## Anatomy of a protein kinase

**Figure 1.**
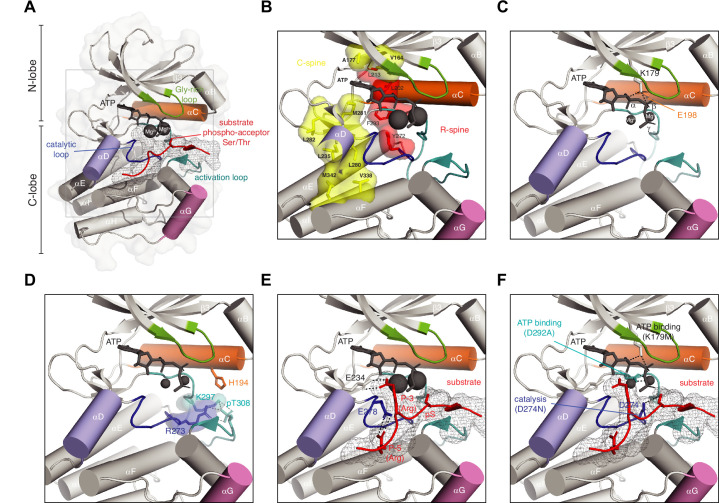
Anatomy of a protein kinase. (**A**) Basic anatomy of a protein kinase. Important elements in catalysis and regulation are highlighted in color: glycine-rich loop (green), αC helix (orange), αD helix (purple), αG helix (pink), activation loop (teal), catalytic loop (blue), substrate peptide (red) (**B**) Regulatory (red) and catalytic (yellow) spines of a protein kinase that define the active conformation. Residue numbering according to Akt1 (PDB ID: 4ekk). (**C**) Conserved salt bridge between lysine in strand β3 and glutamate in αC helix that defines the active kinase conformation. (**D**) Network of hydrogen bonds that stabilizes the ordered conformation of the activation loop in Akt1 and anchors it to the surface of the kinase domain. (**E**) Substrate recognition by Akt1. Substrate peptide derived from GSK3β (gray mesh, red cartoon) makes specific interactions with conserved glutamates in the kinase domain of Akt1 via arginine side chains in the P-3 and P-5 positions. Residues C-terminal to the phospho-acceptor residue participate in an antiparallel beta-sheet interaction with the activation loop. (**F**) Ways to make an inactive kinase. Mutation of the β3 lysine (K179M), which abrogates ATP binding; mutation of the DFG aspartate (D292A), which abrogates magnesium and ATP binding; mutation of the catalytic aspartate (D274N), which prevents polarization of the substrate hydroxyl and blocks catalysis.

A prerequisite for understanding protein kinase regulation in the context of complex signaling networks is knowledge of the structure of the kinase domain and the catalytic mechanism of phosphoryl transfer. In this section, we provide a structural and mechanistic framework for understanding kinase function. The kinase domain is typified by a bi-lobal fold comprising two sub-domains, the so-called N- and C-lobes (Figure 1A; Knighton et al., 1991). ATP (black), together with two magnesium ions, binds in the cleft between the N- and C-lobes, positioning its γ-phosphate for transfer onto the serine, threonine, or tyrosine side chain of a protein substrate (red). The glycine-rich loop (green) helps to organize the γ-phosphate for phosphoryl-transfer. Substrate binding and recognition are governed by the αD helix (purple) and activation loop (teal). Finally, the αG helix (pink) mediates a wide array of protein-protein interactions that control kinase domain dimerization (Haling et al., 2014; Levina et al., 2022; Park et al., 2019; Patel et al., 2011), *trans*-autoinhibition (Patel et al., 2011; Reinhardt et al., 2023), *trans*-autophosphorylation (Levina et al., 2022; Pirruccello et al., 2006), binding of regulatory subunits (Kim et al., 2007; Lei et al., 2000), substrate recognition (Dar et al., 2005; Komander et al., 2008), and the recruitment of protein phosphatases (Song et al., 2001).

The active conformation of the kinase domain (Figure 1B) is characterized by the assembly of hydrophobic ‘spines’ that have been defined as the catalytic C-spine (yellow) and the regulatory R-spine (red) (Kornev et al., 2008; Kornev et al., 2006), and a conserved salt bridge between a lysine in strand β3 and a glutamate in the αC helix (Figure 1C; Huse and Kuriyan, 2002). The catalytic spine is completed by the adenine moiety of ATP and links nucleotide binding to the orientation of the αC helix (orange) and, thereby, to the correct positioning of the γ-phosphate for phosphoryl-transfer (Endicott et al., 2012). In addition, it ensures the stable formation of the αD helix (purple), required for substrate recognition.

Some kinases, such as Akt/PKB, are regulated by a disorder-to-order transition of their activation loop upon its phosphorylation (Huse and Kuriyan, 2002; Yang et al., 2002b). The phosphorylated activation loop is stabilized by a network of hydrogen bonds such that it docks against the surface of the C-lobe (Figure 1D), thereby creating a surface to which the substrate binds. Mutation of residues that coordinate the phosphate group results in loss of kinase activation and has been implicated in human disease (George et al., 2004). Examples of kinases which are not regulated by phosphorylation of their activation loop include the dystrophia myotonica protein kinases (DMPKs), which exhibit the active conformation constitutively in the absence of phosphorylation (Elkins et al., 2009; Heikkila et al., 2011; Komander et al., 2008; Truebestein et al., 2015; Yamaguchi et al., 2006).

Substrate recognition is achieved by the recognition of a short linear motif containing the phospho-acceptor serine, threonine, or tyrosine residue. In the AGC kinase family, here exemplified by Akt, recognition involves a pair of salt bridges between the kinase domain and the substrate (Figure 1E). A conserved glutamate or aspartate residue at the beginning of helix αD (purple) coordinates the guanidinium group of an arginine side chain at position P-3 in the substrate (red), while a glutamate in the catalytic loop (blue) similarly hydrogen bonds to an arginine in the P-5 position (Yang et al., 2002a). A recent systematic screening of the substrate specificities of all human serine/threonine kinases provides a useful resource for kinase biologists (Johnson et al., 2023). Additional elements outside of the consensus recognition motif that drives the specific interaction of kinase-substrate pairs are particularly well characterized for the mitogen-activated protein kinases (MAPKs) (Miller and Turk, 2018; Sheridan et al., 2008).

Phosphoryl-transfer is intrinsically a dissociative elimination-addition reaction in which nucleophilic attack by the hydroxyl group of the substrate on the phosphorous atom of the terminal γ-phosphate of ATP generates a transition state (Endicott et al., 2012; Lassila et al., 2011). Kinases achieve their remarkable catalytic rate enhancements (up to 3 × 10^5^ fold) in three main ways: positioning of the substrate (described above, Figure 1E), increasing the nucleophilicity of the substrate, and overcoming electrostatic repulsion (Lassila et al., 2011). Crystallographic evidence for a transition state has been obtained in the case of PKA co-crystallized with ADP and aluminum fluoride (Madhusudan et al., 2002). Most recently, the structure of a PKA-product complex obtained by neutron diffraction has revealed the protonation of one of the phosphoryl oxygens on the product peptide and its consequent rotation away from the catalytic site towards the bulk solvent (Gerlits et al., 2019). This finding is consistent with an earlier study suggesting that protonation of the product may trigger its release (Gerlits et al., 2015).

From a mechanistic understanding of the kinase reaction, there are a number of ways to inactivate a protein kinase. The first, and most commonly employed, involves mutation of the lysine in strand β3 that coordinates ATP (Figure 1F). Lysine is more often than not replaced with alanine, though some studies have employed arginine (for the preservation of positive charge) or methionine (close to isosteric with lysine). Some studies have shown, however, that lysine substitutions may display residual activity, significant activation loop autophosphorylation, and altered specificity (Eyers et al., 2005; Haydon et al., 2003). Less commonly employed is the mutation of the aspartate of the DFG (Asp-Phe-Gly) motif in the activation loop to alanine (Figure 1F). Both mutations result in the loss of ATP binding, which invariably leads to loss of kinase activity. These mutations, however, sometimes have unintended consequences that arise from the loss of protein stability (Iyer et al., 2005), for which the experimentalist should be aware. This can lead to protein degradation, exposure of regulatory domains, or changes in subcellular localization (Liljedahl et al., 2001; Lučić et al., 2016; Verbeek et al., 2008). The best way to inactivate a protein kinase is to mutate the catalytic aspartate to asparagine (Figure 1F), since it is isosteric with aspartate and permits native side chain interactions while still inactivating the kinase.

Conversely, many studies make use of phosphomimetic substitutions to constitutively activate a protein kinase. However, it is important to distinguish between the chemical properties of the carboxylic acid side chain of aspartate and glutamate, (which carries a single negative charge at physiological pH), and the native phospho-amino acid (in which the phosphate carries two full negative charges). Additionally, their hydrogen bonding capacities are considerably different and, whilst the aspartate side chain is approximately the same length as phosphoserine, glutamate is one carbon longer and may not be capable of making the same interactions. This is particularly relevant in the case of tyrosine kinases, where it is not possible to faithfully mimic phosphotyrosine, although many studies have employed glutamate substitutions. In the same vein, alanine or phenylalanine substitutions used to prevent kinase activation may have unintended effects that are unrelated to the loss of phosphorylation, due to a deficit in hydrogen bonding intrinsic to the substituted amino acid. When designing site-specific mutations to derive mechanistic insight, consideration of protein structure and function is indispensable, irrespective of the target enzyme.

## A conceptual framework for kinase regulation

As intracellular transducers of cellular information, kinases need to be exquisitely responsive to upstream inputs that tune their activity accordingly. This regulatory potential is strongly related to the conformational flexibility previously described that enables kinases to interconvert between an inactive state and an active state, upon which substrate can be phosphorylated with high efficiency. A multitude of signaling inputs can trigger reversible switching between these states. This process of conformational switching requires activating and inhibitory forces, which may come from within the protein kinase itself (*cis*) or from a second protein (*trans*). In the *trans* situation, this may be another protomer of the same molecule (*trans*-auto) or a different protein altogether. Within this framework, the following regulatory mechanisms and features can be combined to describe the regulation of all protein kinases.

### Activation loop conformation

The activation loop has the highest sequence divergence (Modi and Dunbrack, 2019) and the greatest structural flexibility (Nolen et al., 2004) in comparison to the rest of the kinase domain fold. The simplest way to suppress kinase activity is the stabilization of an inhibitory conformation of the activation loop. Subsequent acquisition of the active conformation allows the formation of a high-affinity substrate binding site and organizes the catalytic machinery.

### Activation loop phosphorylation

The conformation of the activation loop can be regulated by phosphorylation, as previously described (Figure 1F). This phosphorylation event can be catalyzed in *cis*, if a kinase molecule is capable of modifying its own activation loop, or in *trans*, either by a second protomer of the same kinase (*trans*-autophosphorylation) or by an upstream kinase (*trans*-phosphorylation). The requirements for autophosphorylation in *cis* and *trans*, as well as their functional implications, are different and will be discussed in more detail later.

### Steric occlusion

Another regulatory mechanism is to block access of the substrate binding cleft. Sometimes referred to as steric occlusion, this can be achieved in *cis* with an inhibitory domain or motif that is encoded on the same polypeptide chain, or in *trans*, either by a second protomer of the same kinase or by a different protein.

### Allostery

Finally, all kinases depend on the precise organization of the catalytic machinery for their activity (Endicott et al., 2012). Allosteric regulation is induced by interactions distal to the catalytic site that can be either inhibitory or activating, and can be mediated both in *cis* and in *trans*. Since almost all kinases are controlled by one or more regulatory domains, it is worth emphasizing that the study of full-length kinases is necessary for a complete understanding of all aspects of kinase structure, function, and conformational dynamics.

In the next two sections, we will discuss well-characterized examples of each of the mechanisms described above, with the goal of illustrating conceptually different solutions to the control of kinase activity.

## Kinase inhibition in *cis* and *trans*

**Figure 2.**
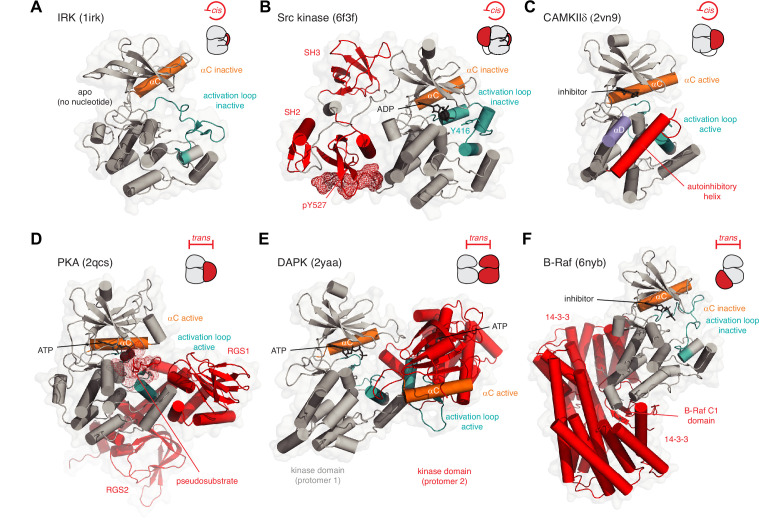
Kinase inhibition in *cis* and *trans*. (**A**) Inhibition of insulin receptor kinase (IRK) in *cis*. The activation loop (teal) of IRK adopts a conformation in which it prevents the binding of ATP and displaces the αC helix into an inactive conformation. PDB ID: 1irk. (**B**) Inhibition of Src tyrosine kinase in *cis*. The regulatory SH3 and SH2 domains (red) help maintain the kinase domain (gray) in an inactive conformation. The activation loop (teal) adopts an inactive conformation in which Y416 is sequestered in an unphosphorylatable conformation and the nucleotide-binding site is occupied by ADP. The phosphorylated C-terminal tail of the kinase domain (pY527, red mesh) binds intramolecularly to the SH2 domain, thereby inhibiting its activation. PDB ID: 6f3f. (**C**) Inhibition of CAMKIIδ in *cis*. A C-terminal autoinhibitory helix (red) in CAMKIIδ occupies the substrate binding surface. PDB ID: 2vn9. (**D**) Inhibition of PKA in *trans*. In the absence of cAMP, the regulatory subunit of PKA (red) binds to the catalytic subunit and inserts its N-terminal pseudosubstrate segment (red mesh) into the substrate binding cleft. PDB ID: 2qcs. (**E**) Inhibition of DAPK in *trans*. Face-to-face dimerization of DAPK (one protomer of DAPK in red) blocks substrate binding. PDB ID: 2yaa. (**F**) Inhibition of B-Raf in *trans*. Binding of 14-3-3 proteins (red) to phosphorylated B-Raf traps its C1 and kinase domains in an autoinhibited conformation in which the kinase domain of Raf is incapable of forming the back-to-back dimer required for its activation. PDB ID: 6nyb.

Keeping kinases inactive in the absence of an activating stimulus is critical for high-fidelity, low-noise signal transduction. Nature has evolved a plethora of mechanisms by which to maintain protein kinases in their inactive conformations when their activity is not required. Relief of these inhibitory interactions is coupled to the acquisition of kinase activity at the right time and place in the cell. At the simplest level, a kinase may be autoinhibited by its own activation loop adopting an inactive conformation. This mechanism has been proposed to regulate a number of receptor tyrosine kinases (RTKs), including the insulin receptor kinase (IRK), in which the inactive conformation of the activation loop is incompatible with ATP binding (Hubbard et al., 1994; Figure 2A). We will revisit this structure later in the context of activation loop autophosphorylation. The Src family of tyrosine kinases also adopts an autoinhibited conformation in which the activation loop both plugs the catalytic cleft and sequesters the tyrosine which undergoes autophosphorylation (Figure 2B). This conformation, which is promoted by intramolecular assembly of the kinase domain with regulatory Src homology 2 (SH2) and Src homology 3 (SH3) domains (Sicheri et al., 1997; Xu et al., 1997) and stabilized by ADP (von Raußendorf et al., 2017), also maintains the αC helix in an inactive conformation in which the conserved lysine-glutamate salt bridge is broken. A homologous inactive conformation is also adopted by the Tec kinases (von Raußendorf et al., 2017; Wang et al., 2015). Individual members of the Src kinases have evolved additional regulatory mechanisms, including phosphorylation of the C-terminal tail in the case of Src and Hck (Sicheri et al., 1997; Xu et al., 1997) (red mesh, Figure 2B), and binding of the N-terminal myristoyl group to a hydrophobic pocket on the C-lobe of the kinase domain in the case of Abl (Hantschel et al., 2003; Nagar et al., 2003). This latter mechanism has recently been exploited with an allosteric inhibitor of Abl (Schoepfer et al., 2018) to treat chronic myelogenous leukemia caused by the breakpoint cluster region (BCR)-Abl fusion protein (Hughes et al., 2019; Wylie et al., 2017).

Other kinases maintain their inactive conformation by steric occlusion of the substrate binding cleft, a mechanism that is employed in both *cis* and *trans*. The Ca^2+^/calmodulin activated kinases (CAMK), for example, are maintained in an inactive conformation by a C-terminal *cis*-autoinhibitory helix that occupies the catalytic cleft (Figure 2C), thereby blocking substrate access (Goldberg et al., 1996; Rellos et al., 2010; Rosenberg et al., 2005). The nematode Twitchin kinase (Titin kinase in humans), also a CAMK, is similarly autoinhibited by a C-terminal helix (Hu et al., 1994; Mayans et al., 1998). In an analogous fashion, PKA is inhibited in *trans* by interactions with its regulatory chain (Figure 2D), which presents a pseudosubstrate peptide to the catalytic cleft (Kim et al., 2007). Recently, a homologous pseudosubstrate motif in the cytoplasmic tail of the G protein-coupled receptor Smoothened has been shown to regulate PKA activity in the Hedgehog signaling pathway (Happ et al., 2022). In the CAMKs, binding of Ca^2+^/CaM to the autoinhibitory helix is required to displace it from the catalytic cleft (Goldberg et al., 1996), while binding of cAMP to the PKA regulatory subunit elicits conformational changes that displace it from the catalytic subunit (Kim et al., 2007).

The diversity of inhibitory mechanisms is further exemplified by members of the death-associated protein kinases (DAPKs), which form face-to-face dimers (Figure 2E) that occlude the catalytic clefts of each protomer (Patel et al., 2011; Simon et al., 2016). Binding of Ca^2+^/CaM to the DAPKs is required for kinase activation (Simon et al., 2016). Recently, an analogous, *trans*-autoinhibited dimer has been reported for the related protein kinase D (PKD) (Reinhardt et al., 2023). Finally, the binding of accessory proteins can regulate kinase activity without occluding the active site (Figure 2F). B-Raf is maintained in an autoinhibited conformation by a dimer of 14-3-3 proteins that binds to two phosphorylated residues in its C-terminus. This creates a cradle that buries the regulatory cysteine-rich domain (CRD) between the 14-3-3 proteins and the B-Raf kinase domain (Park et al., 2019). This protective cradle prevents the back-to-back dimerization of B-Raf that is required to organize its catalytic machinery and drive the phosphorylation of MEK (Park et al., 2019; Rajakulendran et al., 2009; Wan et al., 2004). In summary, a wide array of mechanisms, often employed in combination, serves to acutely regulate the activity of the catalytic domain of protein kinases.

## Kinase activation in *cis* and *trans*

The autoinhibition of protein kinases by their regulatory domains or other protein factors is mirrored by mechanisms that promote their activation. Like inhibition, activation can be accomplished both in *cis* and *trans*.

**Figure 3.**
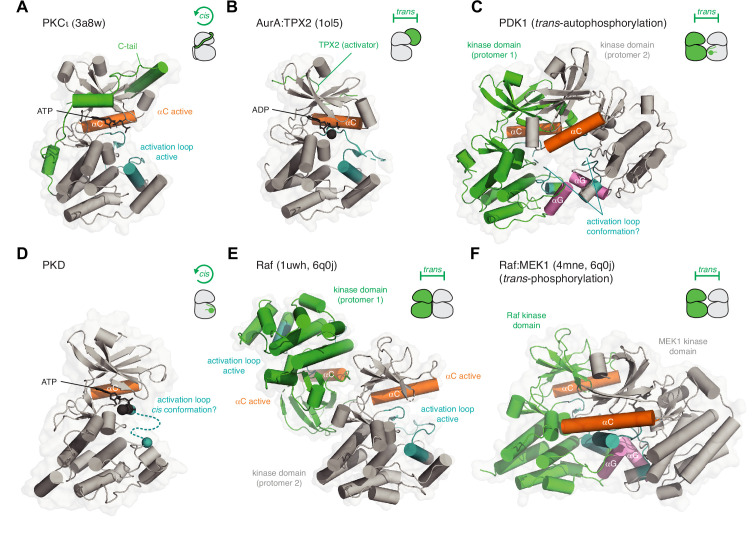
Kinase activation in *cis* and *trans*. (**A**) Activation of PKCι in *cis*. The C-terminal AGC-specific extension of the kinase domain activates PKC by inserting various short linear motifs into regulatory pockets on the surface of the kinase domain. PDB ID: 3a8w. (**B**) Activation of AurA by TPX2 in *trans*. TPX2 inserts a hydrophobic motif into a pocket on the back side of the kinase domain N-lobe in an analogous manner to the hydrophobic motif of PKCι. PDB ID: 1ol5. (**C**) Activation of PDK1 by *trans*-autophosphorylation. Dimerization of the kinase domains via their αG helices (pink) drives *trans*-autophosphorylation of Ser241 in its activation loop. AlphaFold prediction. The mechanism of the reaction and conformation of the activation loop (teal) remains to be determined. (**D**) Activation of PKD1 by *cis*-autophosphorylation. Dissociation of an inactive face-to-face dimer of PKD1 kinase domains leads to activation loop *cis*-autophosphorylation. The mechanism of the reaction and conformation of the activation loop (teal) remains to be determined. (**E**) Activation of B-Raf by back-to-back dimerization of its kinase domains (*trans*). The back-to-back dimer interface stabilizes the active conformation of the αC helix (orange) and activation loop (teal) of each protomer. PDB ID: 1uwh, 6q0j. (**F**) Activation of MEK1 by Raf-mediated phosphorylation of its activation loop in *trans*. B-Raf and MEK1 form a heterodimer via their αG helices, homologous to the dimerization of PDK1. The mechanism of the reaction and conformation of the activation loop(s) (teal) remain to be determined. PDB ID: 4mne, 6q0j.

The AGC kinases are characterized by an approximately 50 amino acid C-terminal extension to their kinase domain that contains a number of *cis*-regulatory elements (Kannan et al., 2007). The tail inserts a series of short linear motifs into regulatory pockets on the kinase domain to stabilize the active conformation (Figure 3A). One such motif is a hydrophobic motif, which, when inserted into a hydrophobic pocket on the N-lobe of the kinase domain, promotes the acquisition of the active conformation of the αC helix and concomitant ordering of the activation loop on the surface of the kinase domain (Pearce et al., 2010; Yang et al., 2002b). Structural and biochemical studies have revealed that the conformation of the tail can be regulated by phosphorylation (Hauge et al., 2007), phosphomimetic amino acids (Takimura et al., 2010; Yang et al., 2002b), additional regulatory domains that co-assemble with the kinase domain (Elkins et al., 2009; Heikkila et al., 2011; Komander et al., 2008; Lodowski et al., 2003; Yamaguchi et al., 2006), or separate protein co-factors that stabilize the interaction of the tail with the kinase domain (Devroe et al., 2004; Parker et al., 2020; Pearce et al., 2010). Recently, a hydrophobic motif was identified in phosphoinositide-dependent kinase 1 (PDK1), the only AGC kinase thought not to contain this regulatory element (Levina et al., 2022). Here, however, the tail has been implicated in the allosteric *trans*-autoactivation of a second protomer of PDK1 in the context of a phosphatidylinositol-3,4,5-trisphosphate (PIP_3_)-bound dimer.

A similar mode of regulation controls the activity of the Aurora kinases, AurA and AurB, also in *trans*. In AurA, targeting protein for Xklp2 (TPX2) inserts a hydrophobic motif into the same pocket on the kinase domain that the AGC kinases use (Figure 3B; Bayliss et al., 2003). The allosteric activation of AurA by TPX2 leads to its subsequent activation by activation loop autophosphorylation (Eyers et al., 2003). In AurB, the IN-box segment of the inner centromere protein (INCENP) fulfills a similar function (Sessa et al., 2005). Conceptually, the activation of the Aurora kinases in *trans* by TPX2 and INCENP is the same as the classical allosteric activation of cyclin-dependent kinases by their cognate cyclins (Wood and Endicott, 2018).

Many protein kinases are activated by the phosphorylation of a conserved amino acid in their activation loop. How they acquire this modification, however, varies and is still, in many cases, controversial. Conventionally, activation loop auto-phosphorylation occurs in *trans*. Such a reaction depends on the transient association of two kinase domains in a dimer and the presentation of the activation loops, as substrates, to the catalytic site of each opposing protomer. Structural evidence for such a mechanism has been obtained for a number of kinases and will be discussed in more detail later. For now, this reaction is illustrated by the kinase PDK1, which forms a transient, face-to-face dimer (Figure 3C) upon binding to membranes containing the lipid second messenger PIP_3_ (Levina et al., 2022).

Whether kinases can perform activation loop phosphorylation in *cis* has long been debated and remains controversial. Contradicting evidence for both *cis* and *trans*-phosphorylation mechanisms has, for example, been reported for AurA (Dodson et al., 2013; Zorba et al., 2014), the checkpoint kinase, Chk2 (Cai et al., 2009; Dodson et al., 2013; Oliver et al., 2006), IRAK4 (Cheng et al., 2007; Ferrao et al., 2014), the insulin receptor (IR) kinase (Frattali et al., 1992; Shoelson et al., 1991; Wu et al., 2008; Yamada et al., 1992), and PKR (Dey et al., 2014; Dey et al., 2005; Mayo et al., 2019), among others. We will revisit some of these cases in more detail later. Models of *cis*-autophosphorylation have been proposed for GSK3ß (Cole et al., 2004), DYRKs (Lochhead et al., 2005), and more recently, PKD (Cobbaut et al., 2018; Reinhardt et al., 2023).

PKD is autoinhibited in *trans* and activated by *cis*-autophosphorylation (Figure 3D; Reinhardt et al., 2023). It has previously been argued that kinases lacking an HRD motif in their catalytic loop (so-called non-HRD kinases) are not activated by activation loop phosphorylation (Johnson et al., 1996; Nolen et al., 2004). Nevertheless, the biochemical characterization of PKD, a non-HRD kinase instead containing an HCD motif, shows its activation by phosphorylation. Molecular modeling and mutagenesis in PKD further demonstrated that the missing arginine in the HCD motif is replaced by a different arginine residue in the activation loop, whereby the side chain reaches into exactly the same position as the canonical arginine in HRD kinases (Reinhardt et al., 2023). Although the mechanism of autophosphorylation remains to be determined for PKD, mass spectrometry unambiguously demonstrates that the reaction occurs exclusively in *cis* (Reinhardt et al., 2023).

Finally, kinase domain dimerization may also lead to allosteric autoactivation. Protein kinase R (PKR), inositol requiring enzyme 1 (IRE1), PKR-like ER kinase (PERK), and NimA-related protein kinase 7 (NEK7), require back-to-back dimerization to induce their activation by autophosphorylation (Cui et al., 2011; Dar et al., 2005; Dey et al., 2005; Lee et al., 2008; Richards et al., 2009). Whether these kinases can accomplish the autophosphorylation of their activation loops in *cis* or require higher-order oligomerization is, however, still the subject of debate (Belyy et al., 2022; Korennykh et al., 2009; Mayo et al., 2019). As discussed earlier in the context of autoinhibition, B-Raf activation depends on the formation of a back-to-back dimer (Figure 3E; Park et al., 2019; Rajakulendran et al., 2009; Wan et al., 2004). The Raf dimer is critical for the activity of B-Raf against its substrate kinase MEK, which forms a heterodimer with the kinase domain of B-Raf that is homologous to the PDK1 homodimer (Levina et al., 2022). Although the precise mechanisms of PDK1 *trans*-autophosphorylation (Figure 3C) and *trans*-phosphorylation of MEK by Raf (Figure 3F) are still not clear, mutagenesis of the αG helix-mediated dimerization interface impairs both PDK1 and MEK activation (Haling et al., 2014; Levina et al., 2022). Finally, kinases of the Inhibitor of kappa B kinase (IKK) family, including IKKε and TANK-binding kinase 1 (TBK1) form constitutive dimers in which the kinase domains are arranged in a back-to-back configuration by a helical stalk domain and a ubiquitin-like domain (Larabi et al., 2013; Tu et al., 2013; Xu et al., 2011). Activation loop autophosphorylation has been proposed to be achieved in an analogous manner to the unfolded protein response (UPR) kinases IRE1 and PERK by higher-order oligomerization (Zhang et al., 2019; Zhao et al., 2019).

## Interpreting biochemical evidence for *cis* and *trans* autophosphorylation

**Figure 4.**
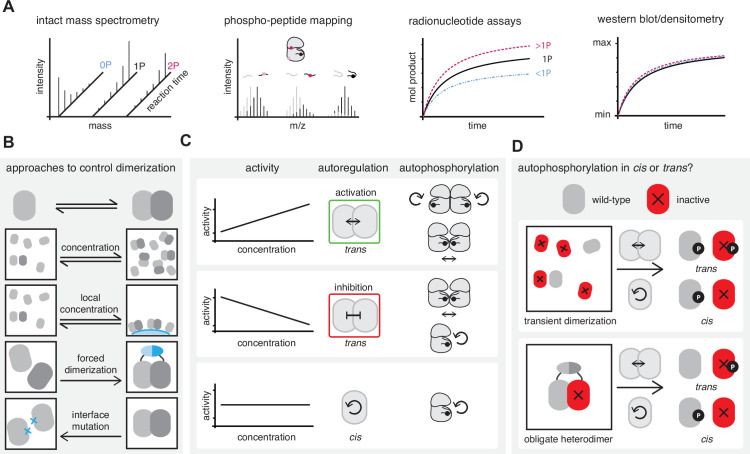
Interpreting biochemical evidence for *cis* and *trans* autophosphorylation. (**A**) Common methods to assay autophosphorylation and their capacity to differentiate stoichiometric mono-phosphorylation (black), from sub-stoichiometric (blue) or super-stoichiometric (magenta) phosphorylation. (**B**) Common approaches to manipulate the monomer-dimer equilibrium of a kinase population in vitro to investigate its effect on autophosphorylation. (**C**) Hypothetical outcomes of dimerization in a kinase autophosphorylation assay. If autophosphorylation increases or decreases with increasing dimerization the reaction is autoactivated or autoinhibited, respectively, in *trans*. Both *cis-* and *trans*-autophosphorylation can be regulated in *trans*. An autophosphorylation reaction that is unaffected by dimerization is not subject to *trans*-autoregulation and likely proceeds in *cis*. (**D**) Catalytically inactive kinases can be phosphorylated in *trans* by an active kinase, but not in *cis*. This can be used to discriminate the reaction mode.

In order to obtain a detailed mechanistic understanding of the autophosphorylation reaction, it is important to perform rigorously controlled biochemical experiments and to interpret the results according to the limitations of the chosen method. Here, we provide an overview of the strengths and weaknesses of the most commonly used readouts of protein phosphorylation (Figure 4A).

### Intact mass spectrometry

Intact mass spectrometry is a powerful tool for the precise and quantitative measurement of protein mass. Modern, well-calibrated instruments are precise to less than 1 Da in mass ranges up to 100 kDa and sometimes even up to 200 kDa (Donnelly et al., 2019). Site-directed mutations and covalent modifications, including common post-translational modifications such as phosphorylation, can be deduced from the mass shifts of the unmodified protein, as well as the stoichiometry of modification. In a more general sense, intact mass spectrometry is an essential quality control step that provides confidence in the interpretation of biochemical results (Elsner et al., 2019; Levina et al., 2022; Pokorny et al., 2021; Reinhardt et al., 2023; Truebestein et al., 2023; Truebestein et al., 2021; Truebestein et al., 2015; von Raußendorf et al., 2017). Intact mass spectrometry does not, however, provide site-specific information and is limited by the quality and complexity of the sample. When performed under non-denaturing conditions, native mass spectrometry can capture non-covalent protein adducts such as ligand-bound species (Bennett et al., 2022; Byrne et al., 2016; Smith et al., 2017), protein-lipid interactions (Agasid and Robinson, 2021), and even protein complexes (Mehmood et al., 2015).

### Tandem mass spectrometry

Phospho-peptide mapping of digested protein samples by tandem mass spectrometry (MS2) can be used to quantify the stoichiometry and specificity of protein phosphorylation. Combined with intact mass spectrometry, this permits an unbiased, site-specific, and quantitative description of the material that is used in biochemical experiments. Tandem mass spectrometry is limited by the ionization efficiency of the peptides resulting from proteolytic cleavage and care must be taken to ensure coverage of the relevant sites. While trypsin cleavage is most commonly employed in such studies, the sequence surrounding a phosphorylation site may not be amenable to trypsin cleavage. In these cases, proteases with different cleavage specificities can be effectively employed to provide coverage of those sites. The ionization properties of phosphorylated peptides can differ from the respective unphosphorylated peptide, so caution should be exercised in interpreting the absolute ratio of the two species. In addition, phosphorylation can hinder proteolytic cleavage, leading to erroneous conclusions regarding the degree of modification. In these situations, a protease with a more suitable cleavage pattern should be chosen.

### Radionucleotide assays

A conventional tool to monitor autophosphorylation reactions is a radionucleotide transfer assay that employs isotope labeled [ɣ- ^32^P] ATP to measure the incorporation of the radioactive ɣ-phosphate into the kinase. By SDS-PAGE, the kinase can be separated from other reaction components or substrates and the kinase-specific autophosphorylation signal can be read out by autoradiography. Careful calibration and background subtraction allows the precise quantification of phosphate transferred during the reaction, but not however, the absolute phosphorylation state. If the absolute phosphorylation state of the starting material is determined, for example by intact mass spectrometry, the average phosphorylation state per kinase molecule can be calculated. Since the assay does not provide information about the phosphorylation site(s), validation of the specificity of the reaction with an unbiased approach like tandem mass spectrometry is required in order to draw robust conclusions.

### Western blotting

Autophosphorylation can also be read out with western blotting using phospho-specific antibodies. These antibodies are typically raised against a phospho-peptide of interest. In the case of multiple phosphorylation sites that overlap the binding epitope of the antibody, these antibodies may only recognize a specific subpopulation of the phosphorylated protein. Conversely, antibodies that have been raised against multiply-phosphorylated peptides may fail to recognize proteins singly phosphorylated on the canonical phospho-site (Waldron et al., 2001). The primary antibody is, in turn, recognized by a secondary antibody, conjugated to a fluorescent label or, more commonly, an enzyme, which amplifies the signal. Western blotting is, at best, semi-quantitative, and at worst, non-specific. While fluorescent secondary antibodies offer a linear dynamic range and, therefore, can be used in a semi-quantitative manner, they are considerably less sensitive than enzyme-coupled secondary antibodies. Conversely, enzyme-coupled secondary antibodies are extremely sensitive and the dynamic range is far from linear. Since the signal readout is usually optimized automatically by chemiluminescent imagers that integrate the signal over time to obtain the best dynamic range for the image, results obtained by western blotting are almost never comparable. Finally, care must be taken in the validation of antibodies, many of which are not specific and are contributing to a reproducibility crisis in molecular biology (Frohner et al., 2020; Pillai-Kastoori et al., 2020; Schüchner et al., 2020). Unlike radionucleotide transfer assays, western-blotting detects phosphorylation irrespective of its origin and is, therefore, suitable for evaluating changes in the phosphorylation state of a sample, when properly calibrated with loading controls. Ideally, the recognition of phosphorylation by phospho-specific antibodies should be cross-validated by tandem mass spectrometry.

The palette of phosphorylation read-outs is further complemented by a variety of semi-quantitative, site-agnostic SDS-PAGE-based approaches including phospho-tyrosine specific western blotting, electrophoretic mobility shift assays, and Phos-tag gels (Nagy et al., 2018). Each of these techniques provides qualitative evidence of phosphorylation, but with limited or no specificity. Irrespective of the employed assay it is advisable to include a kinase-inactive negative control in order to be certain that the signal detected originates from the intended source.

An example of the successful combination of complementary techniques is the characterization of FGFR1 autophosphorylation (Lew et al., 2009). The authors monitored the autophosphorylation of five sites using radionucleotide assays and then discriminated the dynamics of each phosphorylation state in the population by time-resolved intact mass spectrometry. To obtain a more detailed picture of the site-specific dynamics of autophosphorylation, careful mutagenesis was combined with electrophoretic mobility shift assays.

Models of kinase autoregulation are commonly based on the dimerization dependence of the reaction (Figure 4B). Depending on the intrinsic propensity of a kinase to dimerize, the monomer-dimer equilibrium can be manipulated in different ways, the simplest of which is to change the concentration (Cobbs et al., 1989; Cole et al., 2004; Lee et al., 2008; Yamada et al., 1992; Zorba et al., 2014). If the affinity of dimerization is weak, which is desirable for transient interactions, dimerization can be induced by locally concentrating reaction partners on a surface (Chung et al., 2019; Levina et al., 2022; Wang et al., 2015; Zhang et al., 2006) or using either a natively encoded or artificial dimerization domain (Cai et al., 2009; Elsner et al., 2019; Parrini et al., 2002; Reinhardt et al., 2023). If the structural details of the respective dimer are known, mutagenesis of the interface can disrupt dimerization (Haling et al., 2014; Levina et al., 2022; Pike et al., 2008; Rajakulendran et al., 2009; Reinhardt et al., 2023; Wybenga-Groot et al., 2014).

The observation of a concentration-dependent increase in kinase activity is usually interpreted as a bi-molecular reaction in *trans* (Figure 4C). However, this cannot distinguish between autophosphorylation in *trans* (face-to-face), autophosphorylation in *cis* (back-to-back), or even *trans*-autophosphorylation via the face-to-face interaction of back-to-back dimers, as has been proposed for the UPR kinase IRE1 (Korennykh and Walter, 2012; Korennykh et al., 2009). A dimerization-dependent decrease in activity is indicative of autoinhibition in *trans* (Reinhardt et al., 2023). Autophosphorylation reactions that are not influenced by dimerization are not subject to any regulation in *trans* and, as such, likely proceed in *ci*s (GSK).

The gold standard way to discriminate between *cis*- and *trans*-autophosphorylation is to test whether an active kinase can phosphorylate an inactive copy of the same kinase (Figure 4D). This type of experiment can also be performed in the context of an obligate, artificial heterodimer. An important consideration in the design of such experiments is that the autophosphorylation motif of some kinases is highly similar to the consensus substrate recognition motif and likely prone to *trans*-autophosphorylation at high concentrations, irrespective of whether this is the physiological mechanism.

## Interpreting kinetic evidence for *cis-* and *trans*-autophosphorylation

*Cis*- and *trans*-autophosphorylation are commonly discriminated between on the basis of their respective kinetics. In this section, we evaluate the power of enzyme kinetics to discriminate between these reactions, taking into account the limitations of the reductionist experimental setups commonly employed. For simplicity, the scenarios presented do not consider the influence of regulatory elements outside of the kinase domain and assume that ATP and magnesium are present in excess, such that their concentrations can be considered constant during the reaction.

**Figure 5.**
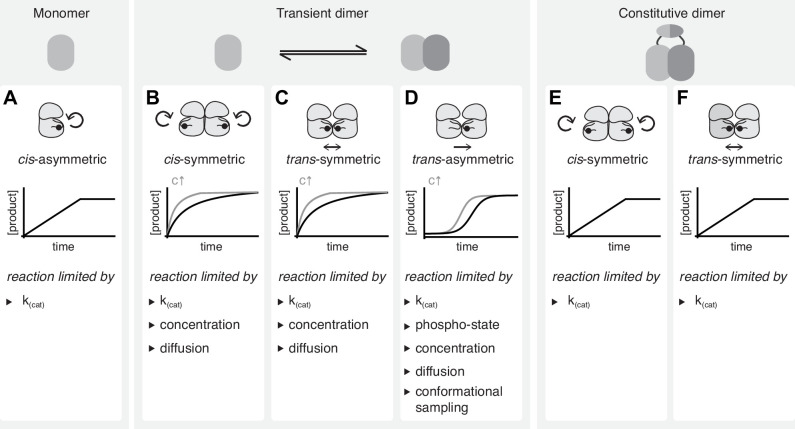
Interpreting kinetic evidence for *cis-* and *trans*-autophosphorylation. (**A**) Kinases that do not dimerize can undergo autophosphorylation only in *cis*. These asymmetric reactions don’t require collision events and are limited only by their intrinsic catalytic rate. They result in linear reaction kinetics. (**B**) Autophosphorylation reactions that are dependent on transient dimerization include *trans*-autoactivated *cis-* and *trans-*autophosphorylation. Symmetric *cis*-autophosphorylation is triggered by the collision with a second kinase (assuming that the activating interaction is phospho-state independent) and, therefore, limited by diffusion and concentration, and proceeds with non-linear kinetics. (**C**) Symmetric *trans*-autophosphorylation is dependent on the collision of two unphosphorylated kinase molecules that phosphorylate each other in a reciprocal reaction which is substrate-limited and proceeds with non-linear kinetics. (**D**) In the asymmetric *trans*-autophosphorylation reaction, one kinase molecule (enzyme) stochastically adopts an ordered activation loop conformation and phosphorylates a second copy (substrate) that presents its activation loop. Upon phosphorylation, the catalytic rate of the kinase increases and the substrate is depleted. This leads to an initial lag phase followed by an exponential phase and a plateau. Such reactions are limited by stochastic conformational sampling and the changing catalytic activity of the population. (**E**) In the context of a constitutively dimeric kinase symmetric *cis*-autophosphorylation is limited only by the intrinsic catalytic rate and proceeds with linear kinetics. (**F**) In the context of a constitutively dimeric kinase symmetric *trans*-autophosphorylation is limited only by the intrinsic catalytic rate and proceeds with linear kinetics.

The simplest case is a unimolecular reaction, in which the activation loop is autophosphorylated in *cis*. If an enzyme converts itself into a product the enzyme is also the substrate and the reaction can happen only once per molecule. It is, therefore, independent of concentration, diffusion, or substrate depletion, and proceeds with a constant catalytic rate until all enzyme has been converted into a product. This results in a linear increase of product over time until the reaction stops and the product remains constant (Figure 5A). For this reaction to be useful in the cell, it must be strictly coupled to an inhibitory mechanism that prevents unregulated signaling.

The second case is autophosphorylation in *trans*, which is necessarily a bimolecular reaction in which the enzyme converts the substrate into a product. This reaction is dependent on (a) the association of the enzyme and the substrate, which is dictated by their interaction affinities and their respective concentrations, (b) on the intrinsic catalytic rate of the enzyme, and (c) on the dissociation of the product. In a closed system such as an in vitro kinase assay, the reaction conditions change over time as the substrate is turned into a product. Regardless, *trans*-autophosphorylation reactions can occur in mechanistically distinct ways that influence the kinetics of the reaction.

A *cis*-autophosphorylation reaction that is dependent on allosteric activation by back-to-back dimerization is a bi-molecular reaction, therefore, concentration-dependent, even if the enzyme and the substrate are the same molecules. We designate the reaction symmetric if the collision of two unphosphorylated molecules results in the phosphorylation of both. If the effect of the allosteric activator is independent of its phosphorylation, the concentration of the activator remains constant, while the substrate is depleted over time. In this case, the reaction is limited by both the intrinsic catalytic rate constant and the substrate concentration. The reaction rate, therefore, decays over time (Figure 5B).

The autophosphorylation of an activation loop in *trans* requires the formation of a face-to-face dimer in which the phosphorylation site sits within the dimerization interface. Upon phosphorylation, the activation loop is stabilized in the active conformation and is no longer able to engage in such an interaction. This implies that only homo-dimeric collisions are productive and that the reaction rate decays with substrate depletion over time (Figure 5C).

In an asymmetric *trans*-autophosphorylation reaction, one kinase acts as an enzyme and one as a substrate. In the absence of phosphorylation to begin with, a productive interaction is only possible if one molecule adopts the active conformation while the second presents its activation loop as a substrate. This would presumably occur by stochastic conformational sampling, the efficiency of which limits the reaction by reducing the frequency of productive collisions. Once phosphorylated, the kinase will be stabilized in the active conformation with a consequent gain in catalytic efficiency, which increases the reaction rate until the substrate becomes limiting. This behavior results in sigmoidal kinetics (Figure 5D).

In the context of physiological signal transduction, transient kinase domain dimerization must be associated with a means of locally concentrating reaction partners to overcome the dependence on diffusion which would otherwise be incompatible with rapid signal transduction. Many autoregulated kinases, therefore, encode dedicated dimerization domains. A conceptually interesting scenario is the constitutive dimerization of a kinase domain via a dimerization domain. The dimer can then be regarded as a single molecule, since the two kinases are at near-infinite local concentration, such that their autophosphorylation becomes independent of both concentration and diffusion. Bi-molecular *cis*- and *trans*-autophosphorylation are thereby reduced to uni-molecular reactions with linear kinetics that are only limited by the intrinsic catalytic rate (Figure 5E–F).

In summary, kinetic data alone cannot necessarily discriminate between *cis*- and *trans*-autophosphorylation. Furthermore, we must take care that our readout is site-specific and keep in mind that what is possible to observe with an isolated kinase domain in solution does not necessarily reflect reality in the cell. In the next section, we will consider what can be learned from the high-resolution structures of kinase dimers determined in the context of a 3-dimensional crystal lattice.

## Interpreting crystallographic evidence for dimerization and autophosphorylation

Dimerization of protein kinases, whether for the purposes of autoinhibition or autoactivation, must necessarily be transient and, therefore, the free energy of binding must be low. If the dimer interface is too strong, the kinase will be constitutively trapped in an inhibited or active state, respectively. Therefore, the study of kinase domain dimerization and autophosphorylation is inherently challenging. Structural insights into the nature of dimerization and autophosphorylation have, historically, relied on X-ray crystallography. New advances in cryo-electron microscopy are continually lowering the mass limit of particles accessible for high-resolution structure determination, but individual kinase domains are still at the limits of current technology (Herzik et al., 2019). In silico protein structure prediction tools such as AlphaFold (Jumper et al., 2021; Senior et al., 2020) and Rosetta (Leman et al., 2020) are revolutionizing the field, and are gradually becoming accessible to researchers who are not experienced computational biologists. Crystallography, however, has been extremely successful in deciphering protein structure and has captured many kinases in various dimeric configurations.

Determining a protein structure by crystallography requires the growth of 3-dimensional crystals in which molecules are packed together in a regular, repeating arrangement such that they diffract X-rays in-phase, leading to measurable reflections. When combined with the missing phase information, the intensities of these reflections provide a near-atomic picture of the underlying protein structure. A crystal lattice can be broken down into two parts: (i) the asymmetric unit, which is the minimum non-symmetric unit of the lattice that, with the application of appropriate symmetry operations, can be copied to make (ii) the unit cell, a rhomboid-shaped unit that can be stacked in a repeating array in 3-dimensions. The formation of a crystal lattice requires contacts between (a) the molecules that make up the unit cell and (b) neighboring unit cells. These contacts do not form accidentally, but rely on an empirical combination of solvent molecules that promote the regular packing of molecules together. Since a crystal lattice is entropically unfavorable, the loss of entropy is offset by a combination of enthalpic free energy gain and the gain in entropy caused by the de-solvation of protein surfaces involved in lattice contacts (Rupp, 2009). As such, the underlying structure represents a snapshot of an energetically favorable state under the conditions of crystallization, which has a large potential to misrepresent protein-protein contacts (Krissinel, 2010). This necessitates that interfaces observed in crystal lattices are carefully validated biochemically.

**Figure 6.**
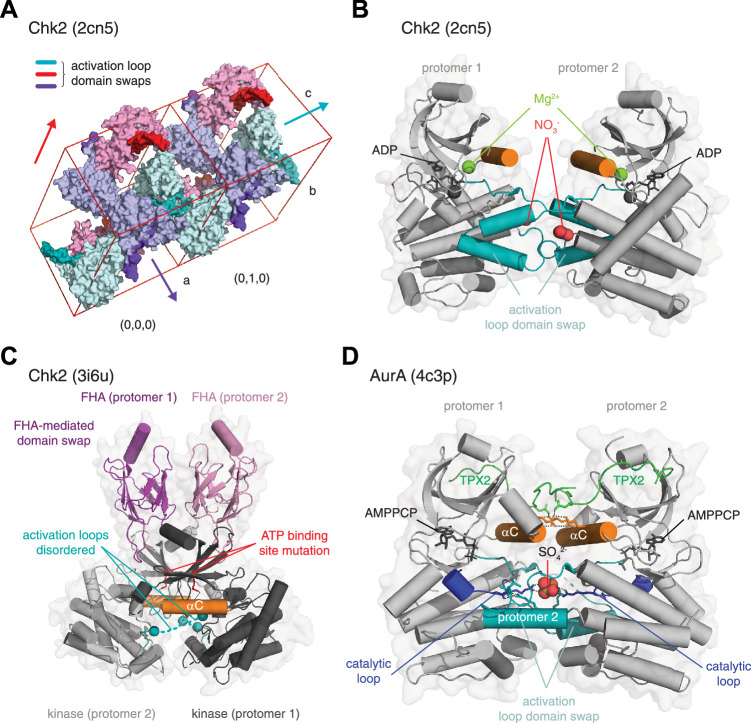
Interpreting crystallographic evidence for kinase dimerization. (**A**) Crystal lattice formation in crystals of the checkpoint kinase (Chk2) domain in complex with ADP. The unit cell contains six molecules (cyan, purple, pink), which form dimers on three twofold crystallographic axes. These are mediated by the extended activation loops (dark color) of Chk2 that undergo activation loop exchange, in which residues 377–397 make either identical interactions with their crystallographically-related partner, or they make interactions with a neighboring molecule in the crystal lattice. Packing of the unit cells and, therefore, propagation of the lattice is mediated in part by the interactions of the activation loops of neighboring molecules. PDB ID: 2cn5. (**B**) Zoom in to the activation loop exchange of Chk2 in the 2cn5 lattice shown in A. The conformation of the Chk2 kinase domain is stabilized by reagents from the crystallization reservoir solution including nitrate (red spheres). One magnesium ion also occupies a non-physiological position in the kinase domain of each Chk2 molecule (green spheres). PDB ID: 2cn5. (**C**) Structure of a domain-swapped Chk2 construct containing both its regulatory FHA (magenta, pink) and catalytic kinase domains (gray, black). The asymmetric unit of the crystal lattice contains two molecules of Chk2 in which a face-to-face dimer of the kinase domains is mediated by a domain swap of the FHA domains of each protomer. The crystallized protein contains a kinase-inactivating point mutation of the β3 lysine (K249R), which abrogates ATP binding. The activation loops (teal) of each protomer are mainly disordered (dashed lines). PDB ID: 3i6u. (**D**) Structure of AurA in complex with TPX2. The asymmetric unit of the crystal lattice contains two molecules of AurA arranged in an asymmetric face-to-face dimer. A sulfate molecule in the center of the dimer stabilizes an exchange of the activation loop of each protomer by making a network of hydrogen bonds with the conserved arginine of the catalytic loop (blue, stick representation). Additional interactions that stabilize the dimer are mediated by electrostatic interactions between the αC helix (orange) and TPX2 (green). AurA was co-crystallized with AMPPCP, but is in an inactive conformation. PDB ID: 4c3p.

The *trans*-autophosphorylation reaction requires the exchange of activation loop segments between opposing protomers in a dimer. By definition, the requirement for phosphorylation dictates that these segments are flexible in their unphosphorylated state and, by extension, that the surface to which they dock in their phosphorylated conformation is not occupied. As such, the activation loop is an exchangeable element that can be used to promote interactions with conserved surfaces on neighboring molecules compatible with crystal lattice growth. There are essentially two flavors of such interactions: one is the docking of the activation loop in *trans* (instead of in *cis*) with the kinase domain of another protomer, and the second is the binding of the activation loop to the substrate binding surface of a neighbor (*trans*). In the absence of conformational restraints imposed by regulatory domains that are often missing, the physiological relevance of these interactions can be hard to deduce from the arrangement of molecules in the crystal lattice. This is further complicated by the fact that activation loop exchange, in which the free energy of binding of the activation loop to its own kinase domain (*cis*) is close to equivalent to that with a neighboring molecule (*trans*), can be used to drive lattice formation by creating a dyad axis of symmetry. Such packing interactions are illustrated by the structure of the kinase domain of Chk2 (Oliver et al., 2006; Figure 6A). The asymmetric unit contains a single molecule of Chk2, which is related to a second molecule in a neighboring asymmetric unit by a twofold axis of symmetry (surface representation of dimers). The unit cell contains six molecules of Chk2, arranged in three equivalent dimers (Figure 6A, light teal, pink, light blue). The two protomers of the dimer interact via the exchange of their activation loops (teal, red, purple-blue) that generates equivalent interactions of residues 368–394 in *trans* as are normally found in *cis*. The exchanged activation loops are stabilized on either side by the N-lobe of a neighboring Chk2 protomer belonging to a different dimer, thereby propagating the lattice in 3-dimensions. The presence of additional compounds from the crystallization solution that mediate specific interactions complicates matters even further. Electron density for two nitrate ions (red) in contact with the activation loop of each protomer was modeled in this structure, while a second magnesium ion (green) was found in a non-physiological location in the N-lobe (Figure 6B).

Activation loop exchanges similar to those described above have been observed in the crystal structures of many kinases, wherein part of the activation loop makes equivalent interactions in *trans* as in *cis* (Lawhorn et al., 2015; Lim et al., 2020; Marcotte et al., 2017; Mayo et al., 2019; Oliver et al., 2007; Oliver et al., 2006; Pike et al., 2008; Taylor et al., 2015; Wu et al., 2019). However, the relative orientation of the kinase domains in these dimers is highly variable and most likely reflects a configuration amenable to lattice formation. Whilst many of these dimers have been interpreted to correspond to intermediates on the path to *trans-*autophosphorylation, equivalent activation loop exchanges have been observed in kinases that do not undergo autophosphorylation, such as DAPK3 (Oliver et al., 2007), for which no physiologically relevant exchange would be required.

Other crystal structures of Chk2, and its yeast homolog, Rad53, offer alternative snapshots. The structure of a longer construct of Chk2, containing its N-terminal regulatory forkhead-associated (FHA) domain (Cai et al., 2009), depicts a dimer in the asymmetric unit, formed by a domain swap between the FHA domains of each protomer (Figure 6C). The intimate association of the kinase domains, coupled with evidence of dimerization in solution and previous biochemical evidence of dimerization-enhanced autophosphorylation (Oliver et al., 2006), led the authors to conclude, logically, that *this* dimer represented the *trans*-autophosphorylation intermediate. Unfortunately, confirmation that this is indeed the case was precluded by absent electron density for the activation loops of each protomer and an ATP-binding mutation introduced into the recombinant protein for crystallization. Consequently, the structure lacks a bound nucleotide and it is not possible to conclude anything definitively about the mechanism of autophosphorylation. The structure of the Rad53 kinase domain is equally problematic: again, a kinase-inactivating mutation was used to facilitate crystallization (Wybenga-Groot et al., 2014). A back-to-back dimer was observed in the asymmetric unit of the crystals, but the authors instead focused on a symmetry-related molecule of Rad53 due to its apparent similarity to the previously reported structure of human Chk2 (Figure 6C; Cai et al., 2009). However, superimposition of one protomer from each dimer shows that, whilst the r.m.s.d. over 156 C_α_ atoms for Chk2 and Rad53 kinase domains is 0.90 Å, this balloons to 11.50 Å over equivalent C_α_ atoms for the second chain. Again, definitive conclusions regarding the mechanism of autophosphorylation are precluded by the absence of electron density for the activation loops. Although not yet supported by an experimentally determined structure, recent in silico modeling and biochemistry on the closely related PKD has revealed a face-to-face *trans*-inhibited dimer (Reinhardt et al., 2023), which begs the question of how Chk2 and PKD could have opposite mechanisms of activation. Further work will undoubtedly be required to address this apparent contradiction.

Finally, AurA is a rich source of conflicting data on its mechanism of autophosphorylation. While kinetic data points towards autophosphorylation in *cis* (Dodson et al., 2013), AurA activation by intermolecular *trans*-autophosphorylation has been inferred from several crystal structures that dimerize via exchange of their activation loops (Lim et al., 2020; Zorba et al., 2014). Zorba et al reported a dimeric, active configuration of AurA, with Asp274 poised for phosphoryl transfer (Zorba et al., 2014). However, careful inspection of the structure reveals that Asp274 is in fact the aspartate of the DFG motif, which coordinates a magnesium ion required for ATP binding. In fact, the catalytic aspartate, Asp256, is not poised for phosphoryl transfer, having been pulled out of position by the interaction of Arg255 with a phosphate ion from the crystallization solution in the center of the exchanged activation loops (Figure 6D). The structure lacks magnesium ions in the catalytic site of both protomers and is not compatible with phosphoryl transfer. It most likely represents a crystallization artifact in which the activation loop exchanged conformation is stabilized by the coordination of the phosphate ion at its center. More recently, AurA has been proposed to be a redox-sensitive switch, inhibited by Coenzyme A (CoA) (Tsuchiya et al., 2020) or activated by increased levels of reactive oxygen species (ROS) during mitosis (Lim et al., 2020). The latter study reports seven crystal structures of AurA in various configurations. Fully reduced, phosphorylated, and monomeric AurA is essentially superimposable with a previous structure of AurA in complex with TPX2 (Bayliss et al., 2003). A second structure, covalently modified on two cysteines by the cacodylate buffer in the crystallization solution, exhibits an activation loop exchanged dimer that bears no similarity to the structure reported by Zorba et al. Two further structures, covalently modified on Cys290 with different compounds, exhibit structures essentially superimposable with the Zorba structure, unsurprising given that the lattice packing is identical in all three cases (although residues 298–309 encompassing Cys290 exhibit a different conformation). Finally, an oxidized, Cys290 disulfide-linked dimer exhibits yet another arrangement of the two protomers. Though autophosphorylation is clearly enhanced in this disulfide-linked dimer in vitro, the kinetics are extremely slow (incomplete after 30 min), an observation which is unexpected for a covalently linked dimer, and which suggests that the conformation of the two protomers in this dimer is not conducive to efficient *trans*-autophosphorylation. Biochemical studies have shown that oxidation inactivates AurA irrespective of activation loop autophosphorylation (Byrne et al., 2020; Tsuchiya et al., 2020).

**Figure 7.**
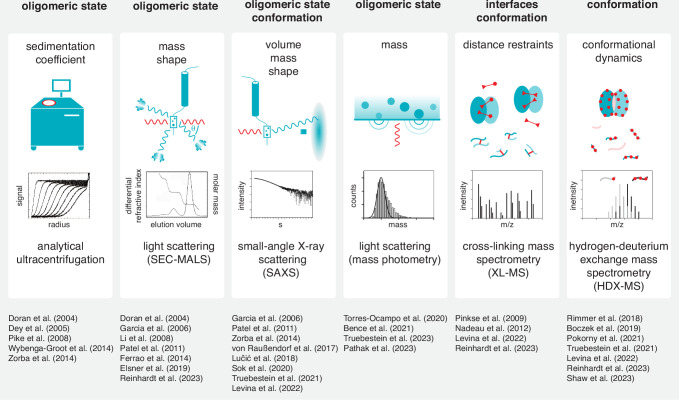
Tools to study kinase dimerization and autophosphorylation in solution. Physical parameters that are measured by each technique are listed alongside the biological questions that can be addressed. Each technique is accompanied by a list of example studies in which the technique has been successfully applied. (Boczek et al., 2019; Doran et al., 2004; Garcia et al., 2006; Hajdusits et al., 2021; Li et al., 2008; Lučić et al., 2018; Nadeau et al., 2012; Pathak et al., 2023; Pinkse et al., 2009; Rimmer et al., 2018; Shaw et al., 2023; Sok et al., 2020; Torres-Ocampo et al., 2020).

The inherent ambiguity in interpreting crystal structures of kinase domain dimers necessitates their validation by other methods. A wide spectrum of tools for the detection of dimerization in solution has been employed, including analytical ultracentrifugation (AUC), size exclusion chromatography coupled to multi-angle light scattering (SEC-MALS), small-angle X-ray scattering (SAXS), mass photometry (MP), cross-linking mass spectrometry (XL-MS) and hydrogen-deuterium exchange mass spectrometry (HDX-MS) (Figure 7). However, the transient nature of dimerization and the limited resolution of each of these methods often precludes definitive corroboration of crystallographic dimers in solution. Furthermore, validation of crystallographic dimers by traditional mutagenesis of the interface is inherently flawed, since the region involved also makes physiologically relevant interactions with its own kinase domain in *cis*, potentially leading to a false positive validation of the interface. In summary, whilst impossible to comprehensively cover them all here, there are many instructive lessons that can be taken from the careful examination and interpretation of a large number of protein kinase structures, and we should be careful not to let our eyes deceive us.

## The enigma of autophosphorylation

**Figure 8.**
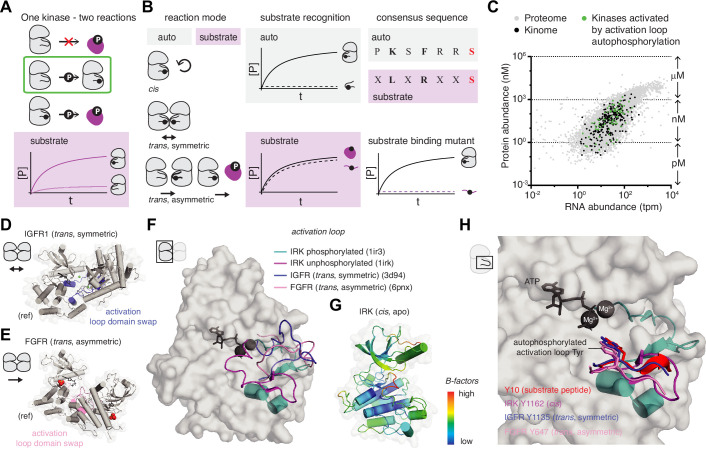
The enigma of autophosphorylation. (**A**) Activation loop autophosphorylation requires the activity of the kinase before it is itself phosphorylated on its activation loop. Logic dictates that the autophosphorylation reaction must, therefore, be mechanistically distinct from substrate phosphorylation. (**B**) Distinct mechanistic aspects of auto- and substrate phosphorylation. Substrate phosphorylation is always an asymmetric *trans* reaction while autophosphorylation can occur in distinct reaction modes. Sequence recognition can vary, depending on the structural context, such that the activation loop is not recognized as a substrate if supplied as a peptide in *trans*. The substrate sequence can also vary between the activation loop and downstream substrates. (**C**) Protein abundance levels were plotted against their RNA transcript levels from a proteome-wide screen in HEK293 cells. The abundance of kinases (black) is in the nanomolar range and relatively low in comparison to the total proteome (gray). Kinases that depend on activation loop autophosphorylation are generally expressed in the nanomolar concentration range, suggesting that *trans*-autoregulated kinases need a dedicated mechanism for dimerization. (**D**) Structure of the kinase domain of insulin-like growth factor receptor (IGFR) in a dimeric, face-to-face configuration in which the activation loop (purple-blue) of each protomer makes symmetric interactions with the catalytic site of the opposing protomer, presenting Y1135 of the activation loop in *trans*. PDB ID: 3d94. (ref = reference molecule). (**E**) Structure of the kinase domain of fibroblast growth factor receptor (FGFR) in a dimeric, face-to-face configuration in which the activation loop (salmon) of one protomer makes asymmetric contacts with the catalytic site of the opposing protomer, presenting Y647 of the activation loop in *trans*. PDB ID: 6pnx. (ref = reference molecule). (**F**) Activation loop trajectories of unphosphorylated insulin receptor kinase (IRK) (magenta), phosphorylated IRK (teal), IGFR in the *trans*, symmetric conformation (purple-blue), and FGFR in the *trans*, asymmetric conformation (salmon), displayed on the surface of the phosphorylated IRK kinase domain. PDB IDs: 1irk, 1ir3, 3d94, 6pnx. (**G**) B-factor plot for IRK kinase domain (apo structure, PDB ID: 1irk). High B-factors for the activation loop indicate that it is the most mobile (and least ordered) region of the kinase domain. (**H**) Zoom-in on the kinase-activation loop interactions observed in: phosphorylated IRK in complex with a substrate peptide (red), unphosphorylated IRK with its own activation loop in *cis* (magenta), IGFR with the activation loop of the opposing protomer in *trans* (symmetric, purple-blue), and FGFR with the activation loop of the opposing protomer in *trans* (asymmetric, pink). PDB IDs: 1irk, 1ir3, 3d94, 6pnx.

The interpretation of crystal structures has been particularly influential in efforts to understand the mechanism of activation loop autophosphorylation. However, before we begin to unpack some of those structures, let us first consider what has come to be known as the ‘enigma of autophosphorylation’ (Beenstock et al., 2016). Simply put, a kinase that relies on the phosphorylation of its activation loop in order to acquire a catalytically competent conformation must somehow be able to perform this reaction without activation loop phosphorylation (Figure 8A). This apparent paradox implies that the autophosphorylation reaction must be mechanistically distinct from the classical substrate phosphorylation reaction. In this final section, we examine some of the models that have been put forward and discuss the various caveats associated with each.

At the most conceptual level, there are essentially three solutions to activation loop autophosphorylation: *cis*-autophosphorylation, symmetric *trans*-autophosphorylation, or asymmetric *trans*-autophosphorylation (Figure 8B). The kinetics of each of these possibilities have been discussed extensively already, so we will restrict ourselves to more conceptual considerations and the interpretation of some example crystal structures that have been proposed to represent the autophosphorylation reaction.

Circumstantial evidence that the autophosphorylation reaction is mechanistically distinct comes from the consensus autophosphorylation motifs of kinases that often don’t resemble their canonical substrate recognition motif (Figure 8B; Beenstock et al., 2016) and are not phosphorylated when presented as a peptide substrate in *trans* (Elsner et al., 2019; Oliver et al., 2006). Experimental evidence that this is the case for at least one kinase has recently been provided by a separation of function mutant of PKD, in which canonical substrate recognition by the conserved glutamate/aspartate in the αD helix (Figure 1A and E) was disrupted (Reinhardt et al., 2023). Whilst mutation of this residue to asparagine was sufficient to completely abrogate phosphorylation of the substrate peptide in *trans*, it had no effect on autophosphorylation in *cis* (Figure 8B).

For the two out of the three solutions that involve autophosphorylation in *trans* and, by definition, necessitate transient dimerization, the concentration of the reactants and their diffusive properties in the cell need to be considered. Kinases are not particularly abundant molecules, with an average concentration in the low-mid nanomolar range (Figure 8C; Hein et al., 2015). Eukaryotic cells are large and crowded places with protein concentrations estimated to be in the region of 200–300 mg/ml (Wiśniewski et al., 2014). By pure diffusion, the average protein with a half-life of 15 hr (relatively long-lived) is estimated to undergo less than 0.1 interactions with another copy of itself (Batada et al., 2004), an estimate which does not even take into account the required geometry for catalysis. As such, any bi-molecular reaction, including *trans*-autophosphorylation, requires the coordinated assembly of two molecules with high temporal and spatial fidelity. Unimolecular reactions, including *cis*-autophosphorylation, in contrast, do not rely on a solution to this search problem. Nature, clearly, has found elegant solutions to trap molecules in the same place. Many receptor tyrosine kinases, for example, have been observed as pre-formed, inactive dimers. Ligand-induced conformational changes transform these receptors into a state in which they are competent of autophosphorylation (Freed et al., 2015; Maruyama, 2015; Moriki et al., 2001). Other kinases can be locally concentrated on a membrane by their specific binding to a lipid second messenger, thereby facilitating autophosphorylation (Chung et al., 2019; Levina et al., 2022; Wang et al., 2015). Finally, subcellular compartmentalization of reactions can restrict the search problem to a much smaller volume (Küchler et al., 2016). What happens next, however, is still controversial.

Early kinetic data indicated that autophosphorylation of IRK could occur in both *cis* (Shoelson et al., 1991; Yamada et al., 1992) and *trans* (Cobbs et al., 1989; Frattali et al., 1992). However, structural and biochemical studies of related kinases, including insulin-like growth factor receptor 1 (IGFR1) and fibroblast growth factor receptor 3 (FGFR3), have since concluded that the reaction occurs in *trans* (Chen et al., 2020; Wu et al., 2008). Indeed, evidence that this reaction can be performed in *trans* has been obtained from a number of crystal structures in which the activation loop of both protomers (Wu et al., 2008) (symmetric, Figure 8D) or just one (Chen et al., 2020) (asymmetric, Figure 8E) is trapped in the active site of the opposing protomer. As can be seen, the symmetric and asymmetric arrangements of the protomers in IGFR1 (Figure 8D) and FGFR3 (Figure 8E) are very different (reference molecule denoted ‘ref’). The promiscuity of lattice contacts is further evidenced by the trapping of IGFR1 in an asymmetric dimer that is again different from the FGFR1 asymmetric dimer, yet still positions the activation loop tyrosine for phosphoryl transfer (Nemecek et al., 2010; Xu et al., 2015). Whether these structural differences reflect fundamental mechanistic differences or simply the energetics of crystal lattice packing is difficult to say, but the implications are quite fundamental. While IGFR1 might accomplish *trans*-autophosphorylation of each protomer simultaneously, FGFR3 would presumably need to go through a two-step reaction mechanism. Such a mechanism has also been proposed for other kinases, including p21-activated kinase (PAK) and Interleukin 1 (IL-1) receptor-associated kinase 4 (IRAK4), based on their crystal structures (Ferrao et al., 2014; Wang et al., 2011). Interestingly, the kinase domain of IRAK1, which apparently does not homodimerize, has been proposed to form heterodimers with phosphorylated, but not unphosphorylated, IRAK4, leading to a model in which IRAK4 phosphorylates IRAK1 (Wang et al., 2017).

Examination of the activation loop trajectories of IGFR1 and FGFR3, shows that they can sample a large conformational space (Figure 8F). Both the phosphorylated, active conformation (teal) and various conformations of the unphosphorylated activation loop have been observed in crystal structures. As we have already discussed, a *cis* conformation of the activation loop has also been observed in IRK (Hubbard et al., 1994) (magenta, Figure 8F and teal, Figure 2A). This structure was interpreted to reflect the inactive conformation of the IRK kinase domain on the basis that the activation loop sterically blocks ATP binding. However, inspection of the B-factors of this structure, which are a measure of the thermal motion of the protein in the crystal lattice, reveals that the activation loop is, in fact, the most mobile and least ordered part of the kinase domain (Figure 8G). Since the protein also lacks a bound nucleotide, the entire conformation of the activation loop may not be physiologically relevant.

Why is this important? Zooming in on the conformation of the activation loop surrounding the phosphorylated tyrosine in the *cis*, *trans* symmetric, *trans* asymmetric, and *trans* substrate structures reveals that the activation loop adopts the same conformation in each case, with the tyrosine poised for transfer of phosphate from ATP to its hydroxyl group (Figure 8H). We can, therefore, conclude from these structures that *cis*, *trans* symmetric, *trans* asymmetric, and *trans* substrate reactions are all stereochemically possible. It is perhaps also not surprising that what are essentially identical interactions can all be observed in the context of a crystal lattice. Does that mean that they can all happen in the cell? The answer to this question is more complicated and depends on the factors that limit each of these reactions, including local concentration and the available conformational space that can be sampled. Many of these mechanisms are surely rooted in strong biochemical evidence, but, once again, we should be careful not to let our eyes deceive us: what we see in a crystal lattice and what we can observe in a test tube do not necessarily reflect reality in the cell.

## Future perspectives

The regulation of protein kinases by activation loop autophosphorylation is an elegant mechanism by which ligand-induced conformational changes can be coupled to kinase activation. However, despite decades of work on numerous kinases, much is still unknown about this seemingly simple reaction. Fundamental barriers to progress include both the difficulty of studying these reactions quantitatively and mechanistically in the complex environment of the cell and, conversely, the pitfalls of the reductionist, in vitro approaches that characterize interactions and biochemistry that are physically and chemically possible, but that may not occur in the context of the native, cellular environment. Spatial confinement, restrictions on the conformational space that can be sampled by a kinase, and local concentration are all tightly regulated in the cell, but challenging to recapitulate in vitro. New in silico tools in structural biology promise much in terms of modeling transient, but specific interactions, while technological improvements in cryogenic electron microscopy are continually lowering the size of structures that can be determined at high resolution. However, we have a collective responsibility to report more quantitatively and more specifically. The reproducibility crisis is particularly grave in the case of antibodies, many of which are poorly validated or have been shown to recognize different targets. While DNA sequencing is nowadays an obligation, we have yet to make the validation of recombinant proteins by mass spectrometry a routine procedure or require its reporting. This is especially important in the context of reversible post-translational modifications, such as phosphorylation, which regulate the conformation, localization, and activity of proteins. Finally, new tools for the spatial arrangement of molecules in vitro on scaffolds that more faithfully mimic the cellular environment are desperately needed. Advances in protein design (Cao et al., 2022; Sahtoe et al., 2022), DNA nanotechnology (Engelhardt et al., 2019; Praetorius and Dietz, 2017), 3D printing of biopolymers (Daly et al., 2021), and label-free, single molecule surface particle tracking Foley et al., 2021; Heermann et al., 2021 have the potential to facilitate more complex in vitro reconstitutions. In summary, new technologies promise much discovery, but hypothesis-driven research compels us to first cast a critical eye over existing bodies of evidence.
